# The Saint Louis World’s Fair — a Physician’s Hostelry

**Published:** 1904-09

**Authors:** 


### The Saint Louis World's Fair — A Physician's Hostelry.

ONE of the great objections raised against expositions of late is the lack of comfortable hotel accommodations at reasonable rates. This is a topic of special interest to physicians who always expect to pay liberally for comfortable quarters, but do not tolerate extortion. For the most part they prefer a hotel to a boarding house, hence are prepared to pay reasonable hotel prices.

This important subject has its bearing, too, on the success or failure of an exposition; therefore, accurate information is most desirable. Physicians would often make a visit to such an exhibition as a part of their summer vacation, could they have the accurate information in advance upon the point in question. We propose to put our readers in possession of sufficient facts bearing on this topic, as far as they relate to the Saint Louis Fair, to enable them to act with intelligence in regard to it.

The Hotel Monticello, situated at the Kingshighway and Pine Boulevard, on the edge of Forest Park, meets all the requirements that we have indicated. It is a modern structure, built of substantial brick, hence is in no sense a temporary hotel. It is well ventilated, heated by steam, with running hot and cold water in every room, baths between each apartment, and is supplied with Bell telephones throughout the house.

A correspondent of a leading American newspaper writing under date of July 12, 1904, of hotels at Saint Louis in general, says of the Monticello in particular:

My hotel was on the great Park Front boulevard of the city, known as Kingshighway. Flanking it on every side were beautiful hotels, overlooking the wide shades and classic comforts of millionaire residences, private parks, and, best of all, the great and beautiful Forest Park. These surroundings made me comfortable at the fashionable, but old-fashioned and delightful Monticello.

This hotel affords its guests free automobile service on their arrival at the Union Station, which is, of course, exceptional, treats them not as strangers, but as friends, and delights every one with its hospitality.

The Monticello is kept on the European plan, and any physician in Buffalo or vicinity who contemplates visiting the fair, can make no mistake in securing accommodation at this most desirable hotel. The location is one of the best, being ten minutes' walk from the fair grounds through Forest Park; there is also an automobile service every 15 minutes, as well as electric trolley lines which keep the guests in constant touch with the great exhibition.

Applications for reservation of rooms should be made to Mr. L. C. Irvine, manager, who will supply prompt information relating to every need of prospective guests.

